# Acceleration of the SPADE Method Using a Custom-Tailored FP-Growth Implementation

**DOI:** 10.3389/fninf.2021.723406

**Published:** 2021-09-16

**Authors:** Florian Porrmann, Sarah Pilz, Alessandra Stella, Alexander Kleinjohann, Michael Denker, Jens Hagemeyer, Ulrich Rückert

**Affiliations:** ^1^Cognitronics and Sensor Systems, CITEC, Bielefeld University, Bielefeld, Germany; ^2^Institute of Neuroscience and Medicine (INM-6) and Institute for Advanced Simulation (IAS-6) and JARA-Institute Brain Structure-Function Relationships (INM-10), Jülich Research Center, Jülich, Germany; ^3^RWTH Aachen University, Aachen, Germany

**Keywords:** FP-growth, pattern mining, spike train analysis, embedded devices, performance optimization, low power, parallel and distributed computing, heterogeneous computing

## Abstract

The *SPADE* (spatio-temporal **S**pike **PA**ttern **D**etection and **E**valuation) method was developed to find reoccurring spatio-temporal patterns in neuronal spike activity (parallel spike trains). However, depending on the number of spike trains and the length of recording, this method can exhibit long runtimes. Based on a realistic benchmark data set, we identified that the combination of pattern mining (using the *FP-Growth* algorithm) and the result filtering account for 85–90% of the method's total runtime. Therefore, in this paper, we propose a customized *FP-Growth* implementation tailored to the requirements of *SPADE*, which significantly accelerates pattern mining and result filtering. Our version allows for parallel and distributed execution, and due to the improvements made, an execution on heterogeneous and low-power embedded devices is now also possible. The implementation has been evaluated using a traditional workstation based on an Intel Broadwell Xeon E5-1650 v4 as a baseline. Furthermore, the heterogeneous microserver platform RECS|Box has been used for evaluating the implementation on two HiSilicon Hi1616 (Kunpeng 916), an Intel Coffee Lake-ER Xeon E-2276ME, an Intel Broadwell Xeon D-D1577, and three NVIDIA Tegra devices (Jetson AGX Xavier, Jetson Xavier NX, and Jetson TX2). Depending on the platform, our implementation is between 27 and 200 times faster than the original implementation. At the same time, the energy consumption was reduced by up to two orders of magnitude.

## 1. Introduction

Increasing evidence from neuroscience suggests that in order to understand the principles of information processing in the brain, it is important to study not only the activity of isolated neurons in response to the environment and behavior, but also to investigate the concerted dynamics of neuronal networks as a whole. With the rapid advancement of electrophysiological recording techniques in the recent decades, scientists are now able to monitor the spiking activity of individual nerve cells in large neuronal populations, enabling the investigation of the dynamics of hundreds of neurons recorded in parallel (e.g., Jun et al., 2017; Brochier et al., 2018; Steinmetz et al., 2018; Juavinett et al., 2019; Chen et al., 2020). The cell assembly hypothesis (Hebb, 1949) postulates that information is represented by interactions within groups of neurons. Signatures of assemblies in the observed dynamics are groups of synchronously active neurons (e.g., Harris, 2005), or spatio-temporal sequences of neuronal activation. Efficient methods to detect and characterize this coordinated activity are in high demand (Quaglio et al., 2018). Such methods need to deal with challenges related to the highly non-stationary spike time series and the statistical complexity of high-dimensional activity patterns, since the number of possible patterns exponentially increases with the number of observed neurons. Several complementary methods have been developed and calibrated in the past (e.g., Grün et al., 2002a,b; Pipa et al., 2008; Gerstein et al., 2012; Lopes-dos Santos et al., 2013; Torre et al., 2013; Russo and Durstewitz, 2017; Diana et al., 2019; Watanabe et al., 2019; Williams et al., 2020). While the nature and underlying assumptions of these approaches differ, they share the need to scale in runtime performance as the number of observed neurons or the length of the recording increases. This holds true, in particular, with an increasing interest to employ such techniques to analyze and validate simulations of large-scale models of neuronal networks (cf., e.g., Trensch et al., 2018; Gutzen et al., 2018) that easily exceed the volume of available experimental data.

One of the state-of-the-art methods to detect spatio-temporal patterns in large sets of parallel spike trains (Quaglio et al., 2018) is *SPADE*^1^, originally proposed by Torre et al. (2013). The method is based on frequent itemset mining (Agrawal et al., 1993). The existing Python implementation of the SPADE method in the Electrophysiology Analysis Toolkit^2^ (Elephant; RRID:SCR_003833; Denker et al., 2018) is able to analyze current data sets of moderate size at relatively high computational cost, making the availability of distributed compute resources mandatory and discouraging interactive exploratory analyses. In this work, we put forward an accelerated version of *SPADE* by optimizing the underlying pattern mining flow using a custom-tailored *FP-Growth*^3^ (Han et al., 2000) implementation to address the need for enhanced scalability and thereby increase the range of data sets for which the method is practically applicable. Additionally, we show that our optimizations enable the execution of *SPADE* on heterogeneous and low-power embedded devices, which is significantly more energy-efficient than the execution on a modern workstation.

Previously, the focus of development efforts related to *SPADE* concentrated on improving or extending the capabilities of the method, which makes this work the first to address performance and energy efficiency. After Torre et al. (2013) developed the concepts for the statistical evaluation of synchronous spike patterns through *FP-Growth*, Yegenoglu et al. (2016) introduced a technique to identify *spatio-temporal patterns* in *massively parallel spike trains* using formal concept analysis (FCA; Ganter and Wille, 1999), extending the detection of patterns from synchronous to spike patterns with delays. In 2017, these approaches were combined by Quaglio et al. (2017). Since the FCA implementation used by Yegenoglu et al. (2016) required significantly more time and computational power, it was replaced by *FP-Growth*. Stella et al. (2019) introduced an extension to *SPADE*, called *3d-SPADE*, which also accounts for the temporal extent of patterns with delays in the significance estimation. The *SPADE* method is explained in more detail in section 2.3.

On a similar path, the *FP-Growth* algorithm used in *SPADE* (Picado-Muiño et al., 2013) was subject to numerous extensions and modifications from a methodological perspective. Picado Muiño et al. (2012) and Borgelt and Picado-Muiño (2013) introduced a version of FP-Growth in continuous time called CoCoNAD, which avoids the need to discretize the input spike train. CoCoNAD was used for benchmarking of artificial data (Picado-Muiño et al., 2013) and analyses of electrophysiological experiments (Torre et al., 2016). Furthermore, CoCoNAD was extended in Borgelt et al. (2015) to account for patterns with selective neuronal participation, or *fuzzy patterns*. When extending the *SPADE* analysis to delayed patterns, it was necessary to resort back to discretizing data (Quaglio et al., 2017).

In contrast to *SPADE*, where performance improvements were never the main focus, several publications focused primarily on improving and accelerating *FP-Growth* through, e.g., parallel or distributed computing. A detailed explanation of the pattern mining and *FP-Growth* related terms used in this section can be found in sections 2.1, 2.2. The first parallel *FP-Growth* variation, called *MLFPT*, was developed by Zaiane et al. (2001). It divides the input database across all available processors and creates a local FP-tree^4^, the data structure used by *FP-Growth*, on each. Afterward, a global header table, a linked list used by *FP-Growth*, is created, linking the different items to their occurrences in local FP-trees. Each processor is assigned an equal portion of the entire itemset on which it performs the pattern mining step.

Chen et al. (2009) developed a parallel *FP-Growth* variant, called *Grided FP-Growth* (*GFP-Growth*), designed to be used on large compute clusters. The main difference to the original *FP-Growth* is that they skip the FP-tree construction by directly mining the conditional pattern bases, sub-databases, created from the FP-tree, using the projection method proposed in Bin and Li (2008). This allows them to split the mining process into independent groups, which can be executed in parallel on any number of compute nodes.

Li et al. (2008) proposed a massively parallel and distributed implementation, called *PFP-Growth*. Their approach is based on *MapReduce* (Dean and Ghemawat, 2004), a programming model for large-scale distributed computing. By dividing the input data into independent *groups*, they can distribute the workload across massive compute clusters without any computational dependencies between the different nodes. In their tests, they achieved nearly linear performance scaling when executing their implementation with a data set consisting of 802,939 web pages on between 100 and 2,500 computers. Zhou et al. (2010) improved *PFP-Growth* by adding load balance features, resulting in a new version they called *BPFP-Growth*. Through proper load balancing during the parallel execution of the pattern mining process, a speedup of 1.5 over the original *PFP-Growth* implementation was achieved. Xia et al. (2018) improved the performance of *PFP-Growth* when processing a massive number of small files on a Hadoop compute platform, resulting in the creation of *MR-PFP-Growth*. Shi et al. (2017) proposed a distributed *FP-Growth* algorithm, using Apache Spark^5^ called *DFPS*, which achieved a significant speedup over *PFP-Growth*.

The previously introduced parallel implementations for *FP-Growth* are designed for use with large data sets, containing a vast number of transactions (1–100 million) and items (more than 10 million), and target large-scale compute clusters with up to several thousand nodes. The algorithms were developed to make pattern mining on these data sets possible in a reasonable time frame. In addition, the use of such massive compute clusters requires good load balancing and fault tolerance so that the computation does not have to be restarted in case a node fails. In contrast, the data sets used with *SPADE* are relatively small, consisting of only a few thousand transactions with, on average, two to three thousand items. Furthermore, while the cited implementations target the parallelization of the baseline *FP-Growth* algorithm, the version developed in this work is custom-tailored for the use in the *SPADE* method. As such, the improved implementation presented here, based around a rather naive approach to parallel and distributed computing of *FP-Growth*, is more suitable for the given problem, as it does not inhibit the portability and can be easily disabled if required. One of the main differences between our implementation and the ones described previously is based on the *filter function*, a part of the SPADE algorithm which significantly reduces the number of patterns mined. It enables us to pursue an implementation approach that would not be possible under normal conditions. Therefore, using code optimizations and minimized overhead, we managed to achieve high performance and high energy efficiency using server- and distributed embedded processors.

The main contributions of this work are as follows.

1. We propose an optimized *FP-Growth* implementation, custom-tailored to the problem presented by the *SPADE* method. A significant performance increase was achieved by incorporating the pattern filtering function used by *SPADE* into the pattern mining. Furthermore, we have implemented parallelization and distributed computing concepts in our customized version of *FP-Growth* to take full advantage of the available hardware.
2. Moving the pattern filtering task into *FP-Growth* resulted in a considerable decrease in memory consumption, to the point where execution on low-power embedded devices is now possible.
3. We evaluated our implementation's performance and showed that a significant performance increase could be achieved with our optimizations compared to the original.

The remainder of this article is structured as follows. In section 2, we first provide an introduction to pattern mining. Subsequently, we introduce the *SPADE* method, in particular, its core algorithm, *FP-Growth*. We identify the bottlenecks of the current implementation and present our optimizations in terms of efficient data handling, memory optimizations, and parallelizations. In section 3, we compare the runtime, energy efficiency, and memory consumption of the original implementation to our optimized solution. For this purpose, we run the optimized version on several different platforms. We demonstrate that our improvements can achieve up to 280 times higher energy efficiency in addition to an acceleration by a factor of up to 200. Finally, in section 4, we discuss the impact of our optimizations on *SPADE's* overall runtime and energy efficiency and present possible future research to improve its performance further.

## 2. Method

In this section, we propose an optimization to significantly accelerate the *SPADE* method used to detect spike patterns in massively parallel spike trains. Therefore, we first discuss the method itself, focusing on the *FP-Growth* algorithm used to identify frequent spike patterns. Afterward, we present our version of *FP-Growth*, optimized for use in the *SPADE* pipeline. By integrating the result filtering step, that had previously been performed separately, directly into the pattern mining process, we achieve a significant performance improvement.

### 2.1. Introduction to Frequent Pattern Mining

In this paragraph, we first give a short introduction into frequent pattern mining and its terminology. Afterward, these concepts are showcased in a small example. Frequent pattern mining refers to the task of identifying reoccurring patterns within large databases. Agrawal et al. (1993) initially introduced this concept to find patterns in large databases of customer transactions, e.g., from large stores or businesses. Such patterns can, for instance, be used to optimize the product placement in a supermarket, as they provide information about products commonly bought together. In the following, the terms used in conjunction with pattern mining and the concept itself are explained in more detail. Most terms reflect the method's origin in purchase analysis, i.e., *item* and *transaction*. Given an itemset *I*, a transaction *T* is defined as a subset of items from *I*. A transaction database *D* is defined as a collection of transactions. A frequent pattern (itemset) is a combination of items within a transaction that reoccurs in one or more different transactions of the same database. The occurrence count of a pattern is called *support S*. There are different ways to limit the number of patterns produced, e.g., by setting a minimum pattern length, i.e., that a pattern has to contain at least *n*-items to be counted or by specifying a minimum occurrence count, i.e., that a pattern has to occur at least *m*-times to be counted. Additionally, there are two unique categories of frequent patterns: closed frequent patterns and maximal frequent patterns. A pattern *P* is considered closed when there exists no superset, i.e., a pattern containing *P* with the same support *S* as *P*. Similarly, a pattern *P* is regarded as a maximal frequent pattern if it has no frequent superset, i.e., there exists no frequent pattern containing *P*.

**Figure 1:**
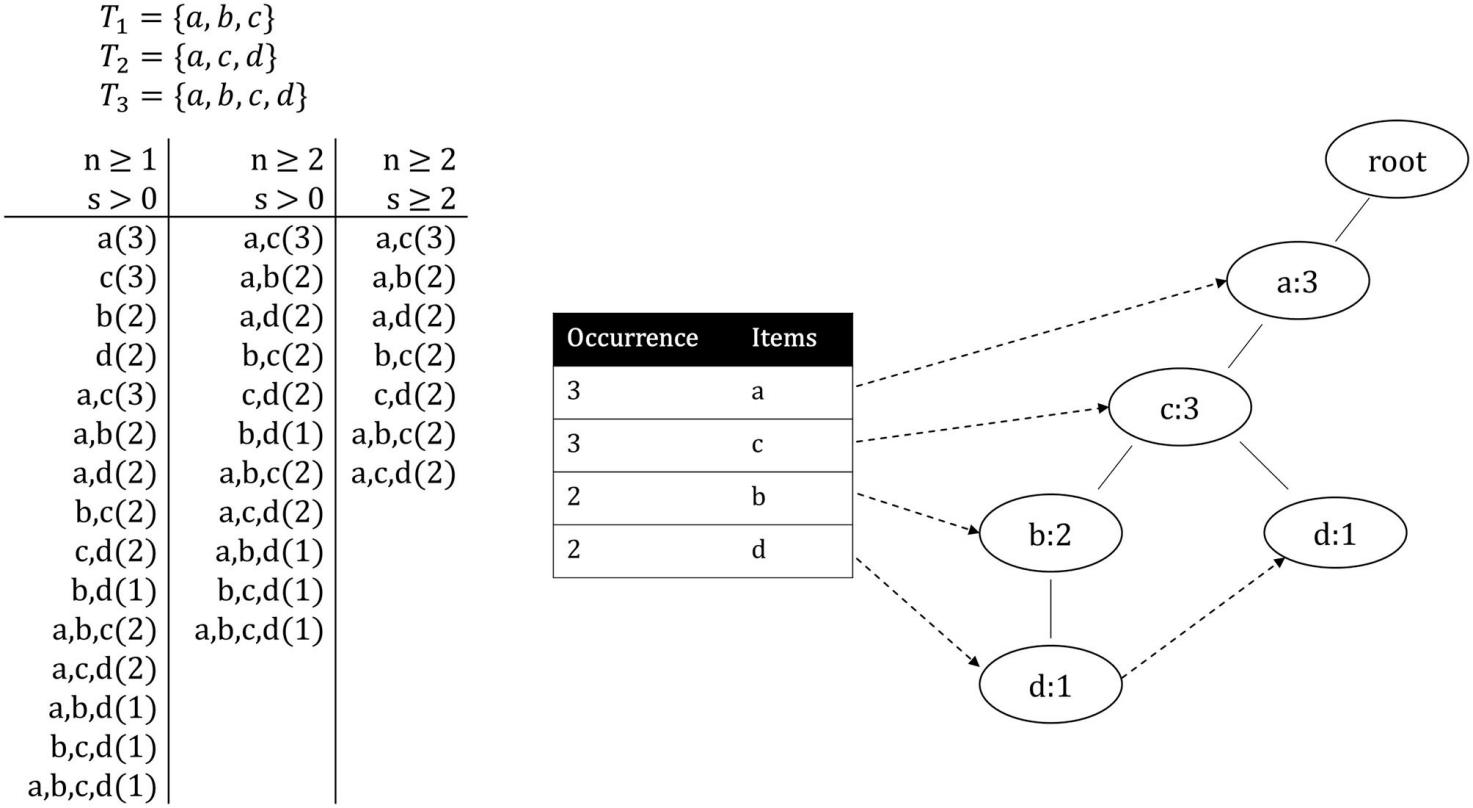
**Left**: All patterns from the pattern mining example presented in section 2.1. **Right**: The header table and FP-tree created from the same example transactions.

The following example showcases the concepts defined above. A pattern *P* is depicted in the form *P* = {*i*_1_, ..., *i*_*n*_}(*S*) with *i* ∈ *I*. Given the itemset *I* = {*a, b, c, d*} and database *D* = {*T*_1_, *T*_2_, *T*_3_} where the transactions are *T*_1_ = {*a, b, c*}, *T*_2_ = {*a, c, d*} and *T*_3_ = {*a, b, c, d*}, without any limitations, 15 frequent patterns can be found in *D*, as shown in Figure 1. Once the minimum pattern length *n* is increased to 2, only 11 patterns remain. If now also a minimum occurrence *s* of 2 is specified, the amount of patterns is reduced to 7. Of these patterns, *a, c*(3), *a, c, d*(2) and *a, b, c*(2) are closed and *a, c, d*(2) and *a, b, c*(2) are maximal frequent patterns.

### 2.2. FP-Growth-Based Pattern Mining

The *FP-Growth* algorithm is a highly efficient method to mine frequent patterns from a transaction database. Other well-known algorithms for frequent pattern mining, such as the *Eclat* (Zaki, 2000) or the *Apriori* (Agrawal and Srikant, 1994) algorithm, perform this task through candidate generation, which has the drawback that it can consume a large amount of memory. *FP-Growth* builds a so-called FP-tree, which contains all information about the relations between different items in all transactions. By traversing this tree and recursively creating so-called conditional sub-trees, it is possible to find all frequent patterns without candidate generation, while also requiring significantly less memory. The algorithm operates as follows. First, it iterates over the entire database to store all unique items and their occurrence in a list *L*, sorted by occurrence. Afterward, all items with an occurrence count below the threshold can directly be discarded. The same applies to transactions that have fewer items than required for the minimum pattern length. Next, the items in each transaction are sorted in descending order based on their occurrence. Subsequently, the actual FP-tree is created by first creating a root-node and sequentially inserting the transactions into the tree. Starting at the root node, for the first item of the current transaction, either a new node is created (if no node for this item exists) or the counter is incremented (if a node exists). This process is repeated for each item in the transaction, always using the newly created node as a base. Once the current transaction has been fully processed, the same process is done for the next transaction, starting once again at the root node. This is repeated until all transactions have been processed and the FP-tree is completed. In parallel to the FP-tree, a header table is built, linking each unique item to its first occurrence in the tree, which then, in turn, links to the second occurrence, and so on. These links are known as *node-links*. The items' order is defined by their occurrence and is equal to the order in the previously created list *L*. The header table and the FP-tree for the example presented in section 2.1 are depicted in Figure 1.

After the FP-tree and the header table are created, the frequent patterns are mined. This is done by iterating over the header table and evaluating the *node-link* for the respective item *i*. If *i* only occurs once within the tree, the frequent patterns can be determined by creating all combinations of *i* with its preceding nodes. Should *i* occur multiple times in the tree, the preceding nodes form the so-called *conditional pattern base* of *i*, from which a sub-FP-tree is created, called *conditional FP-tree* of *i*. The mining process is recursively performed on the conditional tree until all patterns have been mined. Once all patterns for a header table entry have been computed, the same process is repeated for the next entry until the entire header table has been processed, and therefore, all frequent patterns have been mined. It should be noted that there exist no dependencies between the different header table iterations, meaning that they could, in theory, all be performed in parallel. The compute complexity of the *FP-Growth* algorithm depends on the number of items in the header table and the maximum depth of the FP-tree, i.e., again, the number of items. Let *n* be the number of items. Therefore, the complexity of *FP-Growth* is *O*(*n*^2^) (Wicaksono et al., 2020).

### 2.3. Spike Activity Analysis Using the SPADE Method

The *SPADE* method was introduced by Torre et al. (2013) and has since been continuously advanced and improved (Quaglio et al., 2017; Stella et al., 2019). Using *SPADE*, it is possible to detect spatio-temporal spike patterns in parallel spike trains. Spatio-temporal spike patterns are precisely reoccurring delayed sequences of spikes across neurons. They are defined by the times of their occurrences, by the neurons involved, and by the temporal delays between spikes. In order to detect spatio-temporal patterns, *SPADE* employs frequent itemset mining to find reoccurring candidate patterns in the parallel spike train data given as input. The mined patterns are then evaluated for significance by Monte Carlo testing. First, different realizations of surrogate data are generated, which are mined using *FP-Growth* similarly to the original data. Second, patterns detected in surrogates are grouped by shared characteristics, i.e., their number of spikes, duration in time, and number of occurrences, and a *p*-value is estimated for each group. In a third step, candidate patterns are selected according to their *p*-value, correcting for multiple testing. Finally, the set of statistically significant patterns is further reduced by conditionally testing each pair of patterns with common spikes. Within this study, we concentrate on the mining of frequent patterns without taking into consideration the statistical tests.

**Figure 2:**
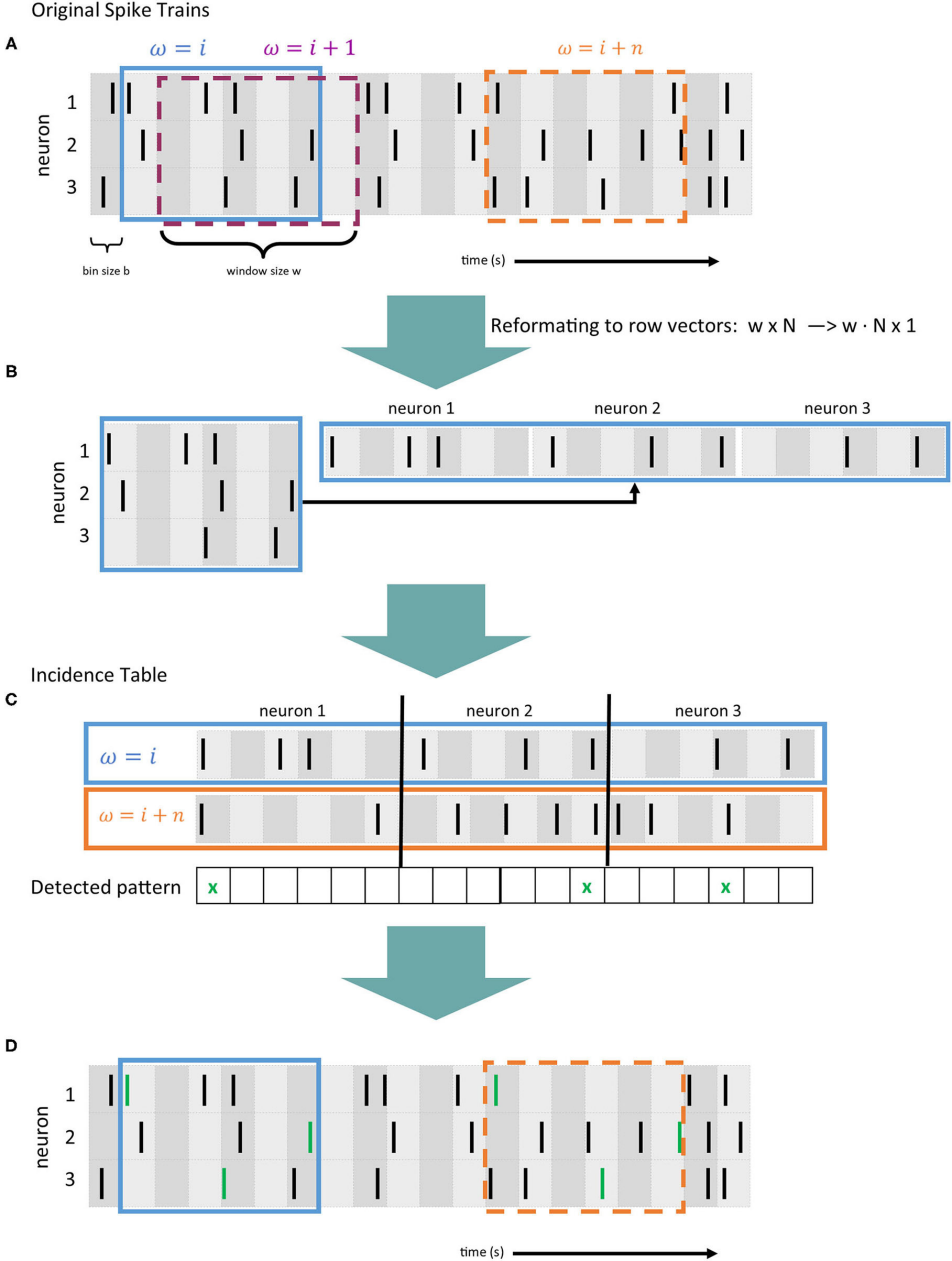
Data preprocessing and evaluation flow of *SPADE* [based on Stella et al. (2019)]. **(A)** Example of 4 spike trains recorded in parallel, where each black line represents a spike. Time is divided into bins (gray vertical areas) of length *b*. A sliding window of size *w* is shifted bin by bin over the data (in blue, purple and orange). **(B)** The window matrix representation is converted to a row vector. **(C)** Simplified visualization of the pattern mining process (also called incidence table), where spikes occurring in the same bin in two window positions (ω = *i* and ω = *i* + *n*) are detected. Coincident spikes across the two windows are indicated with a green cross. **(D)** Representation of the original spike trains as in panel A, where the spike pattern is detected and indicated with green lines.

In terms of required computation, between 85 and 90% of *SPADE's* runtime is spent detecting spike patterns within the parallel spike train data fed into the method. For this, first, the spike trains for all *N* neurons are discretized into *time bins* by segmenting time into small intervals with a bin size *b* of typically a few milliseconds and mapping each spike onto one bin. If two spikes of the same neuron fall into the same bin, they are considered as one spike. This binning technique accounts for small temporal variability that could prevent patterns from being detected. As a next step, in order to detect delayed spike patterns, a sliding window with a length of *w* bins (duration equal to *w* · *b*) is shifted bin by bin over the data (Figure 2A). The quantity ω coincides with the maximal allowed duration of a pattern, calculated as the difference in bins between the first and the last spike. Each window is first provided in a matrix representation with the neurons mapped to the rows and the bins to the columns. For further computation, the matrix is converted to a row vector (cf., Figure 2B). For each element within the window, its position in the vector is calculated as *n* · *w* + *B*, where *n* is the neuron id (row), *w* the length of the window, and *B* the bin id (column). We use ω to denote the index of the window positions (cf. Figures 2A,C). This row vector equals a transaction, as described in section 2.1. The vectors of all windows compose the input data for *FP-Growth* (see section 2.2), the pattern mining algorithm employed by *SPADE*. Figure 2C shows a highly simplified version of the pattern mining process, and Figure 2D depicts the spike trains fed into *SPADE* with the found pattern highlighted in green.

Since typically, a large number of neurons is involved, only closed frequent patterns are kept, while non-closed patterns are rejected (Torre et al., 2013). After the mining is done, the output can still contain repeating patterns caused by the shifting window. A pattern with a duration shorter than the shifting window size will reoccur several times in different windows. Therefore, only those patterns whose first spike occurs in the first bin are kept, and all others are discarded. This can be quickly done, assuming that *P* is the position of the pattern within the row vector by checking if *P* mod *w* = 0 for any of the occurrences of the pattern. Furthermore, a pattern should also contain a minimum number of individual neurons and only occur a maximum number of times to be considered relevant. Patterns with fewer individual neurons or too many occurrences are therefore also ignored. Due to the use of the window and binning, the same neuron can be part of a pattern multiple times, therefore, it is checked, that at least a minimum number of individual neurons are part of the pattern. This entire filtering step is done by applying a custom *filter function* (cf. **1**) to all found patterns, removing a significant portion of them. Of the three filter criteria mentioned, most patterns are discarded when performing the first bin check. Thereby, a large part (typically, between 90 and 100%) of all found patterns are removed. While *SPADE* is in most parts implemented using Python, for the *FP-Growth* algorithm, the highly optimized C-implementation *PyFIM*^6^, developed by Christian Borgelt, is used (Borgelt and Picado-Muiño, 2013; Picado-Muiño et al., 2013).

**Algorithm 1:** Filter function used by *SPADE*

**Input:** The pattern *P*, the support of the pattern *S*, the minimum number of neurons *mn* and the maximum support *ms*.
**Output:** Whether to keep the pattern or discard it.
**function** FILTER_RESULT(*P, S, w, mn, ms*)
**if** *S* > *ms* **then**
**return** *false*
**end if**
*valid* ← *false*
*neurons* ← [] ⊳ Initialize the list of known neurons
*cnt* ← 0
**for each** *e* ∈ *P* **do**
**if** *e* mod *w* = 0 **then** ⊳ Check if the spike occurred in the first bin
*valid* ← *true*
**end if**
$n\leftarrow \frac{e}{w}$ ⊳ Get the neuron id
**if** *n* ∉ *neurons* **then** ⊳ Check if the neuron has already been checked
*neurons*[*cnt*] ← *n* ⊳ Add the neuron to the known list
*cnt* ← *cnt* + 1 ⊳ Increment the counter
**end if**
**end for**
**if** *cnt* < *mn* **then**
*valid* ← *false*
**end if**
**return** *valid*
**end function**

### 2.4. Identification of Bottlenecks

As mentioned in section 2.3, one of the most time-consuming parts of the *SPADE* method consists of the closed frequent pattern mining, using the *FP-Growth* algorithm, and the result filtering. Therefore, we will first analyze the current implementations of the aforementioned parts and identify their respective bottlenecks. Subsequently, in section 2.5, we will present our optimized version, which achieves a significant speedup compared to the original.

**Figure 3:**
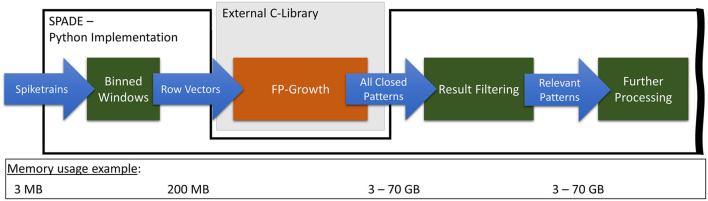
Representation of the original FP-Growth embedding in *SPADE* with special regard to transferred data volumes.

Figure 3 illustrates the current implementation of *SPADE*'s pattern mining flow and its pre-processing steps, on the example of the *movement_PGHF* data set, which is also used during the evaluation (cf., section 3.1). As described in section 2.3, the input spike data is first discretized using binning and the sliding window. Afterward, *FP-Growth* is applied to analyze the resulting row vectors, and all closed patterns are identified. After filtering, only relevant patterns remain and are further processed. For this data set, from 3 MB of spike input data, 200 MB of row vectors are generated and transferred to *FP-Growth*. Depending on the minimum support and occurrence configurations, *FP-Growth* can consume up to 70 GB of memory.

From our analysis of the current state, we identified three main factors for the long runtime of this part of the algorithm. First, a generic *FP-Growth* implementation is used instead of one that is custom-tailored to the problem at hand. Second, all frequent patterns found by the algorithm are sent back to the Python code. Last, the filtering of the results is performed in Python. As noted in section 2.3, the highly optimized C-implementation of the *FP-Growth* algorithm is used in *SPADE*. However, due to the way *SPADE* operates, it does not need all possible closed patterns; it, in fact, only needs a fraction of them. Therefore, using an implementation that mines all closed patterns, as is currently the case, can significantly impact the performance. Furthermore, due to the data structures used internally by the *FP-Growth* implementation, all items of each found pattern have to be mapped back to their original data elements and inserted into a *numpy*-array to be usable in Python. This process can require a significant amount of time and memory and will be referred to as *conversion to Python*. Depending on the number of patterns, this can take several tens of minutes and consume up to 70 GB of memory. Finally, filtering out the repeating patterns takes a long time, as this is done in pure Python, without the assistance of an optimized C or C++ function, which could considerably speed up the process.

### 2.5. Optimized Implementation

We resolved the bottlenecks identified in section 2.4, thereby increasing the performance by several orders of magnitude. This was done by developing a custom C++-based *FP-Growth* implementation, which directly includes the result filtering in an external C++-library.

#### 2.5.1. Custom FP-Growth Implementation With Result Filtering

**Figure 4:**
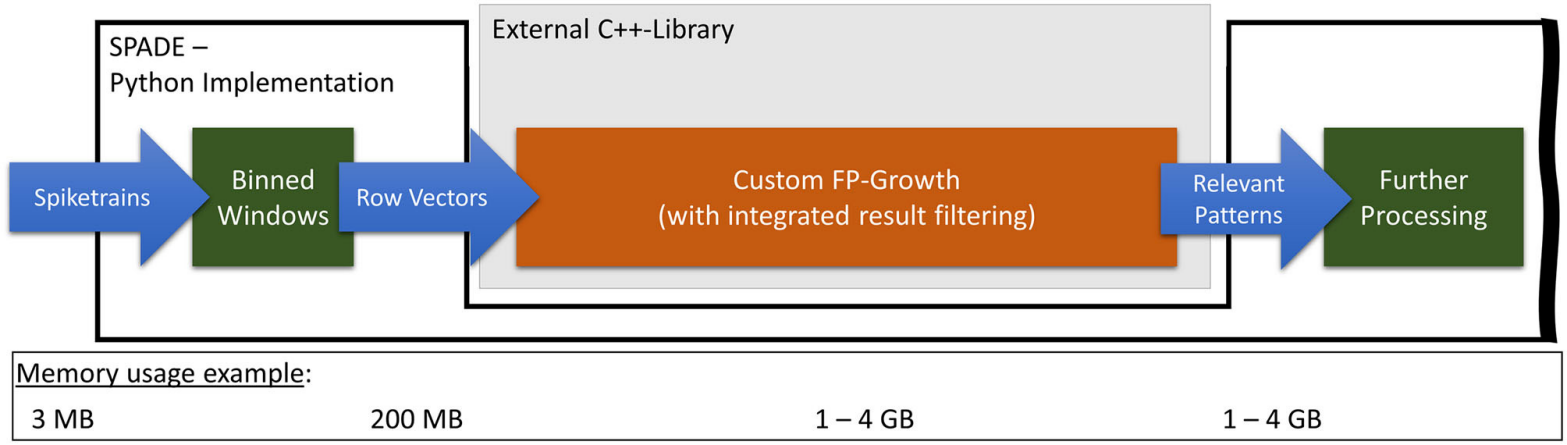
Representation of the optimized FP-Growth embedding in SPADE with special regard to transferred data volumes.

The developed custom C++-based *FP-Growth* implementation is, in parts, based on *PyFIM* by Christian Borgelt. The core implementation of the closed pattern detection, using conditional itemset repositories (Grahne and Zhu, 2003), is entirely adopted from *PyFIM*. There are two significant differences between our version of *FP-Growth* and the general-purpose solution used before. First, the result filter function, applied by *SPADE* to the found closed frequent patterns, is integrated directly into *FP-Growth*. This shifts the filtering from Python to C++, thereby significantly decreasing the runtime and memory consumption, as only a fraction of all patterns needs to be saved. Second, the closed detection is not performed during the pattern mining process but instead afterward. This step was taken because, as mentioned before, the runtime of the closed frequent pattern detection scales with the number of patterns to check. Therefore, integrating the filter function into *FP-Growth* considerably reduces the number of patterns to check for closure. This decreases the runtime of the closed pattern detection and thus results in pattern mining requiring most of the runtime. Furthermore, the implementation for closed frequent pattern detection used in this work cannot be parallelized, in contrast to the pattern mining, which, as noted in section 2.2, can be reasonably easily performed in parallel. In a situation where the closed pattern detection has to be performed on all patterns, i.e., when there is no filter in place, splitting the mining and detection usually either does not affect the runtime or can even increase it. This is because detecting closed patterns is significantly more complex than pattern mining. Figure 4 shows how *SPADEs* pattern mining flow changes when using our optimized *FP-Growth* module. Compared to the original flow, the peak memory consumption was reduced from up to 70 GB down to 4 GB.

#### 2.5.2. Pattern Collector

In our custom *FP-Growth* version, we implemented a pattern collector to efficiently and adequately store the found patterns. It stores the pattern, its length, and support directly in memory. The collector allocates a block of memory each time the previous block is full or the new pattern's size exceeds the remaining space. Additionally, access functions have been integrated to allow for fast iteration over all stored patterns. Furthermore, we directly integrated the pattern filter function into the collector. This way, whenever a new pattern is passed to the collector, it first runs through the filter, and if it is invalid, it is discarded. As a result, only valid patterns are stored, and all others are discarded.

### 2.6. Parallelization and Distributed Computing

As an additional step, we integrated *OpenMP*^7^ into our *FP-Growth* implementation, allowing us to parallelize the pattern mining process across all available CPU-cores, thereby significantly increasing the performance. As mentioned in section 2.2, parallelization of the pattern mining is possible because, when iterating over the header table, all iterations are entirely independent of each other, allowing them to be executed in parallel and in any order. Memory conflicts and potential race conditions were evaded by replicating the internal memory structures for each thread, preventing the threads from affecting each other. However, the closed frequent pattern detection algorithm requires its input patterns to be in an orderly fashion, i.e., the results of the first iteration, followed by the results of the second iteration, and so on. Therefore, we further modified the code to instantiate *n* pattern collector objects, where *n* is the header table's size. This way, each entry in the header table has its own pattern collector to store all found patterns. This allows the closed detector to operate correctly and removes overhead caused by the threading, as all threads no longer share a single pattern collector. Once the pattern mining process is finished, the closed pattern detector iterates over all *n* collector objects and identifies the closed frequent patterns. As mentioned in section 2.5.1, our implementation uses the closed pattern detector developed by Christian Borgelt, which cannot be easily parallelized, as mentioned in section 2.2. Therefore, at the moment, the closed pattern detection is performed sequentially on a single core.

The complete independence of the header table iterations allows for the pattern mining to be performed in parallel on all cores of a local processor and computed in parallel on several compute nodes. For this purpose, we integrated *MPI*^8^ into our application to distribute the workload across different compute nodes. Through the use of the *MPI* execution environment *mpirun*, it is possible to spawn an arbitrary number of processes for a given application. Furthermore, spawning processes is not limited to the local system but can be done on an arbitrary number of remote nodes, e.g., a compute cluster. However, without integrating *MPI*-specific modifications into the code, execution across multiple nodes will only cause each node to run the entire application. Therefore, the *MPI-API* provides a large selection of functions to allow the processes to communicate, i.e., pass messages between each other. Each process possesses a unique identification number, the so-called *rank*. The *rank* will be a number between 0 and the number of processes spawned by *MPI*. In most cases, one process, usually with *rank* 0, collects all results from all processes once they are finished and presents them to the user or continues working with them.

When integrating *MPI* into our code, only a few modifications were necessary. First, the header table loop was modified to start at the *rank* of the current process and stops iterating in steps of one, but instead in steps of size *p*, where *p* equals the total number of processes. This way, each process processes $\frac{n}{p}$ iterations. We equally distributed the workload across all nodes using a round-robin-styled loop to decrease the chance that one process finishes significantly ahead of the others. Finally, after the header table has been processed and all patterns have been mined, all processes except for the root process send their mined patterns, in the correct order, to the root, where they are added to the correct collectors. Afterward, all but the root process terminate, and the root process performs the closed pattern detection and outputs the final results to the user. It should be noted that our distributed approach requires the entire FP-tree to be built on each node, which can take a significant amount of time for large data sets. However, this is not of any concern because due to the nature of the data, the data sets used with *SPADE* are relatively small, causing the FP-tree creation to only take a few seconds.

## 3. Results

In this section, we evaluate the performance, in terms of runtime, memory consumption, and energy efficiency, of our optimized pattern mining flow on several different devices and compare it to *SPADE's* original program flow. Since in this work, we primarily focused on accelerating the pattern mining and filtering, only the runtimes of the associated steps are examined in the following. Therefore, full runtime refers to the total runtime required by all tasks, i.e., pattern mining, data conversion to Python, and pattern filtering. Since in the original implementation, the pattern mining step also included closed pattern detection and data conversion to Python, for the baseline, these steps are not listed separately. Because we have separated these steps in our optimized version, we include the corresponding runtimes. We show that using our optimizations considerably reduces the runtime and memory consumption and noticeably increases energy efficiency, while producing the same results as the original. Furthermore, due to the memory optimizations, it is now possible to perform the pattern mining on low-power embedded devices.

### 3.1. Test Setup

We used different platforms for evaluation. The first platform, serving as a baseline, was a workstation equipped with an Intel Xeon E5-1650 v4 (6 cores running at 3.60 GHz) server CPU and 256 GB quad-channel DDR4 memory, running Ubuntu 16.04. For the other evaluations, we used our RECS|Box^9^ server (Oleksiak et al., 2019), a modular and scalable microserver platform for resource-efficient heterogeneous high-performance computing.

The RECS|Box is a heterogeneous cluster server that allows the user to choose between several computer architectures, network systems, network topologies, and microserver sizes. In this context, a microserver refers to an independent computer-on-module (CoM) that integrates all components (e.g., CPU, memory, IO, and power subsystem) in a small, compact form factor for integration into a server or embedded environment. In contrast to existing homogeneous microserver platforms that support only a single microserver architecture, RECS|Box seamlessly integrates the full range of microserver technologies in a single chassis, including various CPUs as well as accelerators based on FPGAs^10^ and GPUs. Hence, it can be used to easily set up heterogeneous processing platforms optimized for specific application requirements. CoMs are available for all major computing platforms in both low-power and high-performance versions. Like the big-little approach known from mobile processors, this can be used to further increase energy efficiency by dynamically switching, e.g., between 64-bit ARM server processors and 64-bit ARM mobile SoCs^11^ or between different FPGA/GPU devices.

**Figure 5:**
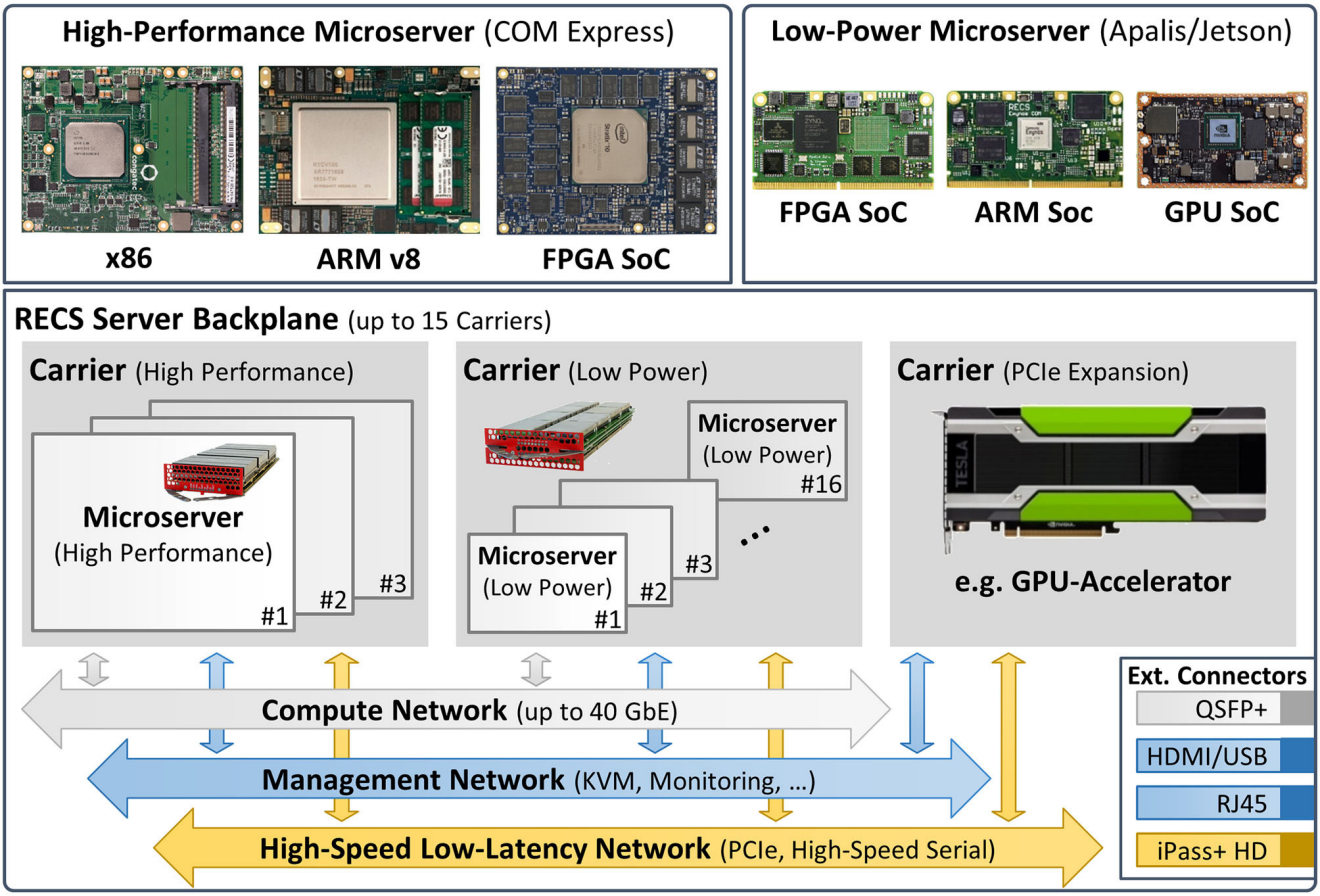
Overview of the RECS|Box hardware platform.

Figure 5 gives a high-level overview of the modular approach used for the design of the RECS|Box system architecture. This modularity guarantees flexibility and reusability and thus high maintainability. Microservers are grouped on carrier boards that support hot-swapping and hot-plugging, similar to a blade-style server. Three different carriers are available: one integrating 16 low-power microservers, one for three high-performance microservers, and one integrating PCIe-based hardware accelerators. All microservers are designed based on well-established CoM form factors^12^, which facilitates the integration of third-party microserver modules into the RECS|Box.

Not only can the platform be individually adapted to the given problem due to its modularity, but it is also able to monitor the power consumption of the individual compute modules very precisely. Furthermore, the installed modules can communicate with each other through high-speed Ethernet over PCI-Express, allowing for fast data exchange, e.g., when performing distributed computing.

For our evaluation, we used high-performance as well as low-power microservers. Firstly, we used a microserver equipped with a HiSilicon *Hi1616* (Kunpeng 916) dotriaconta-core ARM processor (32 cores running at 2.4 GHz) and 64 GB of quad-channel DDR4 memory, running CentOS 7.6, in a dual-socket configuration (resulting in 64 cores/128 GB). In the following, this will be referred to as the *Hi1616* microserver. Next, an *ADLINK Express-BD7*^13^ module, equipped with an Intel Xeon D-1577 (16 cores running at 1.30 GHz) and 32 GB dual-channel DDR4 memory running Ubuntu 18.04 was used. Additionally, we used an *ADLINK Express-CFR-E*^14^ microserver, equipped with an Intel Xeon E-2276ME (6 cores running at 2.8 GHz) and 32 GB of dual-channel DDR4 memory, also running Ubuntu 18.04. Finally, we executed our implementation on three different types of embedded NVIDIA Jetson devices, each running Ubuntu 18.04.

As mentioned above, we also evaluated energy efficiency by measuring each platform's system power consumption during the execution of the test. System power consumption refers to the amount of power consumed by the entire system after the power supply unit (PSU), i.e., CPU, memory, storage, and system accessories. We measure after the PSU because, depending on the unit's quality and overall load, there can be a significant difference between the system's power and the PSU. Using the monitoring features of the RECS|Box, we were able to accurately measure the power consumption of the different devices installed in it. For the workstation, the power consumption was calculated based on continuous voltage and current measurements using a Tektronix MDO4054B-6^15^ oscilloscope in combination with a Tektronix TCP0030A^16^ current probe. Using the TCP0030A probe, it is possible to continuously measure the electrical current of the 12 V power supply with a sampling rate between 500 and 2,500 samples per second. All tests were performed in an air-conditioned room at about 19°C; therefore, the DC gain accuracy of the probe is < 1% (cf. Tektronix, 2006).

For the evaluation, we used neural data extracted from *in-vivo* experimental recordings. In the experiment, a macaque monkey performs a delayed reaching and grasping task, while its neural activity is recorded using a 10x10 electrode array chronically inserted in the premotor and motor cortex (Riehle et al., 2013; Brochier et al., 2018). The experimental protocol is as follows: the monkey is trained to self-initiate the trial by pressing a start button, then to wait for a first visual cue, indicating the type of grip that it has to perform (either precision grip -PG- or side grip -SG-). After a delay period of 1 s, the monkey receives the *GO* signal, together with the information of the amount of force to apply on the object (high force -HF- or low force -LF-). After the monkey has successfully grasped and pulled the object with the correct grip, a reward is given. In this study, we consider session *i140703-001* of Monkey *N* which lasts 1003 s, and consists of 141 correct trials with randomized trial type order (i.e., combinations of grip and force conditions: *PGHF, PGLF, SGHF, SGLF*). Detailed descriptions of this published data set are given in Brochier et al. (2018). For this data, the SPADE method can be used to detect behaviorally-locked spatio-temporal spike patterns, mimicking the analysis performed in (Torre et al., 2016). In this scenario, it is necessary to segment the data in order to perform a time-resolved analysis: we segment trials into six 500 ms long epochs, each related to a behaviorally relevant event of the trial (*start, cue presentation, early delay, late delay, movement, reward*). Identical epochs belonging to the same trial type are concatenated to form a total of 6x4 = 24 data sets to be analyzed with SPADE. In this example, we consider the segment in which the monkey performs the reaching and grasping movement with precision grip and high force (*movement_PGHF*). The data set has a total duration of 22.32 s, consists of 32 concatenated trials, and has 150 units recorded in parallel after pre-processing. We select specifically only single unit activities (SUA) exhibiting signal to noise ratio (SNR) >2.5. Furthermore, a buffer time of 200 ms is inserted between successive trials.

This data set is a typical use case for *SPADE* in both length and number of observed neurons, making it a fitting example to benchmark the performance of the method. When transforming the input data, as described in section 2.3, 3602 transactions with 3,000 unique items were created, using a *bin* size of 5 ms and a window length of 100 ms (20 bins). We divide the analysis into eight different *jobs*, each for a fixed pattern size (number of spikes), starting from 2 and ending at 10+ in steps of one. For each pattern size, the minimum number of occurrences is estimated for optimizing the pattern mining: the distribution of number of occurrences of a chance pattern of fixed size is estimated with a Poisson assumption using the average estimated rate of all neurons. By taking the 95% percentile of this distribution, we estimate the number of occurrences that a non-significant pattern would have under independence, giving us a lower bound for the support in the pattern search. The absolute lower bound for pattern occurrences is fixed to 10. In fact, in a classical use case of the method, we would focus on behavior-specific patterns. Thus, patterns occurring in less than 30% of the total number of trials (~30 trials per combination of epoch and trial type) are not considered. The different configurations of pattern sizes and number of pattern occurrences are described in Table 1. In addition to the configurations, the table also lists the total number of unfiltered frequent patterns found for each job and how many are left after filtering. With these values, the impact of one of our main optimizations, i.e., filtering the patterns directly when they are mined, can be seen very clearly. This significantly reduces the number of patterns to be stored, thus reducing overall memory consumption and reducing the number of patterns fed to the closed detector to a fraction of the original amount. Through filtering, between 90 and 100% of the mined patterns are discarded.

**Table 1:** Configurations of the eight *jobs* used for the evaluation.

**Job**	**Min. occ**.	**Min. spikes**	**Patterns**	**Filtered patterns**
0	88	2	200,971	22,709
1	25	3	16,477,189	1,562,086
2	12	4	246,958,100	8,486,483
3	10	5	424,713,012	398,618
4	10	6	259,915,712	41
5	10	7	109,269,024	0
6	10	8	29,385,509	0
7	10	9	4,637,531	0

### 3.2. Evaluation of the Software Baseline on x86 Server

To determine the runtime, memory consumption, and energy efficiency of the current flow, i.e., create a performance baseline, we executed the latest *SPADE* version (v0.9.0) on the workstation mentioned before, base on an Intel Broadwell Xeon E5-1650 v4. The considerable memory consumption of the baseline flow made execution on the embedded devices impossible. Table 2 depicts the time, in seconds, required for the entire C-based *FP-Growth* flow, the time, in seconds, to perform the result filtering in Python, and the accumulated runtime, in minutes. The runtime for the *FP-Growth* flow includes the data conversion from Python to C, the pattern detection (including closed pattern detection), and the conversion of the results back to Python. Furthermore, the table also lists the peak memory consumption for each *job*. As can be seen, increasingly complex *jobs* can take from a few minutes up to 2 h and consume more than 70 GB of memory. As mentioned in section 2, these high memory requirements are mainly caused by the need to convert all closed patterns (up to 400 million, depending on the *job*) back to Python, where the filtering is performed. The baseline flow required 6 h and 13 min to complete all eight jobs. Based on the workstations' average power consumption of 64.8 W^17^, the entire computation consumed 1.45 MJ^18^.

**Table 2:** Workstation runtime and memory consumption of the implementation currently used in *SPADE*.

**Job**	**FP-growth** **runtime (s)**	**Filtering** **runtime (s)**	**Full** **runtime (s)**	**Peak mem.** **Consumption (GB)**
0	0.9	0.9	1.8	0.4
1	41.4	83.6	125.0	3.3
2	2299.0	1386.9	3685.9	44.0
3	6506.7	2351.9	8858.6	77.5
4	3033.1	1451.7	4484.8	45.8
5	1651.5	647.5	2299.0	21.3
6	1369.1	187.7	1556.8	8.0
7	1336.7	30.8	1367.5	3.7
Sum	16238.4	6141.0	22379.4	

Afterward, we executed our optimized implementation both in single- and in multi-threaded (12-threads) mode. Both runtimes, as well as the peak memory consumption, are depicted in Table 3. As only the *FP-Growth* implementation is affected by threading, there was no noticeable difference in the time required for the *closed frequent pattern detection* or the conversion to Python, so only the results from the single-threaded test are listed. By filtering the results directly during the creation process, it was possible to significantly reduce the peak memory consumption to a maximum of 4 GB. The single-threaded version required 18 min and 11 s to complete all eight jobs, while the multi-threaded version finished all jobs in 3 min and 17 s, making it 114 times as fast as the baseline (see **Table 7**). Regarding energy efficiency, 65 W (70,876 J) and 109.9 W (21,638 J) were consumed in single- and multi-threaded mode, respectively. The multi-threaded implementation achieved an energy efficiency 67 times higher than the current implementation (see **Table 7**).

**Table 3:** Workstation runtime of the optimized implementation in single (ST)- and multi (MT)-threaded mode.

**Job**	**FP-growth** **runtime (s)**		**Closed** **det. (s)**	**Conversion** **to Python (s)**	**Full runtime (s)**		**Peak mem**. **cons. (GB)**	
	**ST**	**MT**			**ST**	**MT**	**ST**	**MT**
0	1.2	0.5	0.0	0.0	1.3	0.6	0.5	0.5
1	14.9	2.4	1.3	1.0	17.2	4.7	1.3	1.3
2	116.4	17.4	13.3	14.3	143.9	45.1	3.5	3.5
3	205.4	32.7	0.5	0.7	206.6	34.0	3.4	3.4
4	195.9	31.3	0.0	0.0	196.0	31.4	1.9	1.9
5	180.9	29.8	0.0	0.0	181.0	29.9	1.8	1.8
6	174.1	25.7	0.0	0.0	174.1	25.8	1.8	1.8
7	171.0	25.2	0.0	0.0	171.1	25.3	1.8	1.8
Sum	1059.8	165.0	15.1	16.0	1091.2	196.8		

### 3.3. Evaluation on RECS|Box for Server Processors

Due to its combined 64 cores running at 2.4 GHz, the *Hi1616* microserver achieved the highest parallel processing speed and overall lowest runtime of all considered platforms (cf. Table 4). In terms of overall runtime, compared to the workstation, it finished all jobs in 57% of the time, with an average power consumption of 123.3 W (13,780 J), 64% of the energy the workstation required. Compared to the baseline, a speedup of 200 was achieved while being 105 times as energy efficient (see **Table 7**). The Intel Xeon D-1577 in the *ADLINK Express-BD7*, on the other hand, required just 2 s longer (3 min and 19 s) than the workstation to finish all jobs. However, the average power consumption of the Xeon D was only 51.1 W (10,164 J), meaning only 47% of the energy was required to finish all jobs compared to the workstation. When comparing the results to the baseline, the Xeon D achieved a speedup of 113 while being 143 times more energy efficient. Finally, the Intel Xeon E-2276ME finished all jobs in the same time as the workstation while requiring on average only 60.3 W (11,887 J), i.e., 55% of the energy the workstation required. Compared to the baseline, a speedup by a factor of 114 together with a 122 times higher energy efficiency was achieved (see **Table 7**).

**Table 4:** Runtime of the multi-threaded implementation on the ADLINK Express-BD7, the ADLINK Express-CFR-E and the HiSilicon Hi1616 microserver.

**Job**	**FP-growth runtime (s)**			**Closed detection (s)**			**Conv. to Python (s)**			**Full runtime (s)**		
	**BD7**	**CFR**	**Hi16**	**BD7**	**CFR**	**Hi16**	**BD7**	**CFR**	**Hi16**	**BD7**	**CFR**	**Hi16**
0	0.9	0.5	0.8	0.0	0.0	0.0	0.0	0.0	0.0	1.0	0.5	0.9
1	2.6	2.2	1.2	2.9	1.1	2.4	1.5	0.6	2.2	7.1	4.0	6.0
2	16.0	16.8	5.0	24.8	10.8	22.9	20.3	8.2	35.6	61.2	35.8	63.7
3	28.0	31.8	8.6	0.8	0.4	0.8	1.1	0.4	1.6	30.0	32.7	11.1
4	27.0	32.0	8.0	0.0	0.0	0.0	0.0	0.0	0.0	27.2	32.1	8.1
5	25.0	30.2	7.5	0.0	0.0	0.0	0.0	0.0	0.0	25.2	30.3	7.6
6	23.8	31.0	7.2	0.0	0.0	0.0	0.0	0.0	0.0	24.0	31.1	7.3
7	23.1	30.4	7.0	0.0	0.0	0.0	0.0	0.0	0.0	23.3	30.5	7.1
Sum	146.4	174.9	45.3	28.5	12.3	26.1	22.9	9.2	39.4	199.0	197.0	111.8

### 3.4. Evaluation on RECS|Box for Embedded Processors

Over the last decade, energy efficiency has become increasingly important in data centers, especially when focusing on cloud computing (Oleksiak et al., 2017). Therefore, we evaluated our implementation's performance and energy efficiency on several embedded devices, namely the NVIDIA Jetson AGX Xavier^19^, the NVIDIA Jetson Xavier NX^20^, and up to four NVIDIA Jetson TX2^21^, each running Ubuntu 18.04. These devices feature low power consumption along with a small form factor and are equipped with between four and eight ARM cores. In addition to its quad-core ARM Cortex-A57 CPU, the Jetson TX2 also possesses a dual-core NVIDIA Denver 2 CPU. In contrast to that, the Jetson AGX and NX use hexa- and octa-core NVIDIA Carmel ARMv8.2 CPUs, respectively. With its 32 GB of DDR4 memory, the AGX Xavier possesses four times as much memory as the Xavier NX and the Jetson TX2, which each are equipped with 8 GB. Changing the power mode makes it possible to adjust the CPU and GPU clock frequency and disable all but one core, e.g., disable the Denver cores on the TX2 and only use the ARM cores or only use four of the eight cores on the AGX Xavier. For our tests, we configured each device to use all available CPU cores at their maximum clock frequency. While each core of the Jetson TX2 and the AGX Xavier can achieve a maximum frequency of 2 GHz, the cores on the Xavier NX are limited to 1.4 GHz when all cores are enabled. Because currently, the GPUs integrated in the devices are not used at all, the GPU frequency was limited as much as possible to reduce power consumption. The achieved performance and energy efficiency values were compared to the original flow and the results from the workstation test presented in section 3.2.

#### 3.4.1. Execution on a Single Device

Table 5 summarizes the runtimes of the three different embedded platforms for all eight jobs. The best performance is achieved by the NVIDIA Jetson AGX Xavier, which completed all jobs in 7 min and 11 s, followed by the Jetson TX2 and the Xavier NX with 9 min, 31 s, and 13 min, 36 s, respectively. Although compared to the workstation, the embedded devices' runtime is between 2.2 and 4.4 times longer, they required significantly less power and consumed overall less energy. The most power was required by the AGX Xavier, which consumed an average of 20.4 W, resulting in an energy consumption of 8,786 J, followed by the Xavier NX with 6.7 W (5,468 J, one-fourth of the workstation), and the least amount of energy was required by the Jetson TX2 with 9.1 W (5,181 J, less than one-fourth of the workstation). Compared to the baseline flow, the embedded devices are between 52 and 27 times faster and between 280 and 165 times more energy efficient (see **Table 7**). These results show that even though the runtime is higher than on a workstation, the use of embedded platforms may be more suitable in situations where energy efficiency is of a higher priority than runtime.

**Table 5:** Runtime of the multi-threaded implementation on all three embedded devices.

**Job**	**FP-growth runtime (s)**			**Closed detection (s)**			**Conversion to Python (s)**			**Full runtime (s)**		
	**AGX**	**NX**	**TX2**	**AGX**	**NX**	**TX2**	**AGX**	**NX**	**TX2**	**AGX**	**NX**	**TX2**
0	1.0	1.7	1.9	0.0	0.0	0.0	0.0	0.0	0.0	1.1	1.9	2.1
1	5.9	10.5	8.7	1.6	2.5	1.9	1.8	2.7	2.8	9.3	15.8	13.6
2	39.3	75.5	55.3	15.0	21.7	19.6	19.3	35.9	31.2	73.6	133.3	106.3
3	71.3	143.8	94.1	0.5	0.7	0.6	0.8	2.2	1.7	72.6	146.8	96.5
4	68.6	137.2	90.6	0.0	0.0	0.0	0.0	0.0	0.0	68.6	137.4	90.8
5	69.6	119.9	89.0	0.0	0.0	0.0	0.0	0.0	0.0	69.7	120.0	89.1
6	68.6	132.5	83.7	0.0	0.0	0.0	0.0	0.0	0.0	68.7	132.6	83.8
7	67.0	128.2	88.9	0.0	0.0	0.0	0.0	0.0	0.0	67.1	128.3	89.0
Sum	391.4	749.3	512.2	17.1	24.9	22.1	21.9	40.7	35.7	430.7	816.1	571.2

#### 3.4.2. Execution on Multiple Devices

In addition to the previous single device execution, we also utilized the *OpenMPI*-based distributed flow described in section 2.6 to run the implementation on up to four NVIDIA Jetson TX2. As mentioned before, only the *FP-Growth* part is accelerated using multi-threading and distributed computing, while everything else is performed sequentially on the root node. Therefore, the runtimes for the closed detection and the conversion to Python are omitted here, as they equal those of the single node execution, depicted in Table 5. Table 6 shows the time required for the *FP-Growth*-based pattern mining, the full runtime of the accelerated section, and the communication overhead. It should be noted that the communication time is part of the *FP-Growth* runtime and is listed separately to show its impact. When looking at the accumulated runtime of the *FP-Growth* part, a noticeable improvement compared to the execution on a single node is visible. For a single TX2, this part took 8:32 min, while, when using two, three, or four TX2, it was reduced to 4:36, 3:15, and 2:32 min, respectively. Two Jetson TX2 significantly outperform the AGX Xavier in terms of runtime and energy efficiency, as the two TX2 only consume 5,986 J 68% of the energy required by the AGX. As only a part of the computation is performed in parallel, an increase in compute nodes will result in a decrease in energy efficiency. However, four TX2 modules are able to finish all jobs in nearly the same amount of time as the workstation (16 s slower) while only consuming 31% (6,670 J) of the energy required by the workstation. Compared to the baseline, the use of between two and four TX2 modules achieved an acceleration by a factor of 66 to 105 and an increase in energy efficiency by a factor of 217 to 242 (see Table 7). Ultimately, the decision to make is whether to decrease the runtime by adding more TX2 nodes, resulting in increasing energy consumption or increasing energy efficiency at the cost of an increased runtime.

**Table 6:** Runtime of the multi-threaded implementation on up to four NVIDIA Jetson TX2.

**Job**	**FP-growth runtime (s)**			**Communication (s)**			**Full runtime (s)**		
	**2 TX2**	**3 TX2**	**4 TX2**	**2 TX2**	**3 TX2**	**4 TX2**	**2 TX2**	**3 TX2**	**4 TX2**
0	1.5	1.7	1.7	0.1	0.2	0.3	1.7	1.8	1.9
1	5.3	4.1	3.8	0.5	0.6	0.9	9.8	0.9	8.3
2	29.5	21.9	17.4	1.5	2.0	2.3	82.7	71.2	70.4
3	52.6	35.3	27.4	0.3	0.5	0.6	55.1	37.8	30.0
4	50.4	33.9	27.9	0.2	0.4	0.5	50.5	34.0	28.0
5	47.4	34.0	23.7	0.2	0.4	0.5	47.5	34.1	23.9
6	45.2	31.5	23.9	0.2	0.4	0.5	45.3	31.6	24.0
7	44.1	32.3	26.0	0.2	0.4	0.5	44.2	32.4	26.1
Sum	276.0	194.7	151.8	3.2	4.9	6.1	336.8	243.8	212.6

**Table 7:** Runtime and energy consumption of all platforms.

**System**	**Power (W)**	**Runtime (s)**	**Energy**		**Improvement over baseline**	
			**Joule**	**Wh**	**Energy**	**Runtime**
Workstation (Baseline)	64.8	22,379.4	1,450,182	402.83	1	1
Workstation (ST)	65.0	1091.2	70,879	19.69	20	21
Workstation (MT)	109.9	196.8	21,638	6.01	67	114
Express-BD7	51.1	198.9	10,164	2.82	143	113
Express-CFR-E	60.3	197.0	11,887	3.30	122	114
Hi1616	123.3	111.8	13,780	3.82	105	200
AGX Xavier	20.4	430.7	8,786	2.44	165	52
Xavier NX	6.7	816.1	5,468	1.52	265	27
Jetson TX2	9.1	571.2	5,181	1.44	280	39
2x Jetson TX2	17.8	336.8	5,986	1.66	242	66
3x Jetson TX2	25.0	243.8	6,093	1.69	238	92
4x Jetson TX2	31.4	212.6	6,670	1.85	217	105

### 3.5. Scalability

We analyzed the scalability of our optimized flow in terms of increased compute power, e.g., multiple NVIDIA *Jetson TX2* and with regards to data sets with varying properties, i.e., longer and shorter recordings as well as recordings with up to 450 neurons. All measurements for both the original flow and our optimized version were performed on the workstation system. For this evaluation, we used four different data sets. First, the entire 1,003 s long recording session of 150 neurons mentioned in section 3.1 was used as a baseline data set to analyze how both implementations handle long data sets. Next, to test the opposite, the first 5 s of the *movement_PGHF* data set were used to analyze the performance when working with short inputs. Finally, to test the effect an increase in neurons has on the runtime, we created two data sets, each with a length of 22.32 s and with 300 and 450 neurons, respectively, by stacking spike trains of the original data set. The total runtime, i.e., *FP-Growth*, filtering, closed detection, and conversion to Python, for each data set and both flows, is given in Table 8. Furthermore, the table lists the runtime as a percentage of the baseline data set's runtime. The original flow was unable to process the *long* recording, as we had to stop it after 30 h after it consumed over 200 GB of memory.

**Table 8:** Full runtime (in seconds) comparison of the original and the optimized flow for different data sets.

**Data set**	**Length (s)**	**Neurons**	**Found patterns**	**Original flow**		**Optimized flow**	
				**Runtime**	**Baseline-%**	**Runtime**	**Baseline-%**
Baseline	22.32	150	10,214,712	22379.4	100%	196.8	100%
Long	1003.00	150	7,097,875	–	–	3052.6	1551%
Short	5.00	150	73,172	89.4	0.4%	4.0	2%
300 Neurons	22.32	300	28,077,304	28257.7	126%	432.2	220%
450 Neurons	22.32	450	64,933,631	64167.5	287%	1241.1	631%

When analyzing the table, it can be seen that when the number of neurons is increased, our implementation does not scale as well as the original. This becomes particularly evident when considering that a tripling of the neurons leads to a more than sixfold increase of the runtime in our version. In comparison, the runtime of the original version did not even triple. In contrast to this, when the recording duration increases or decreases, the scaling is comparable to the original. On the one hand, when the recording time is decreased from 22.32 to 5 s, only 2% of the original runtime was required, i.e., a reduction by a factor of 50. On the other hand, when the recording length is increased by a factor of 45, the runtime increases only by a factor of about 15. The main bottleneck and one of the primary factors for the inadequate scaling of the optimized flow are the closed detection and the data conversion to Python. As seen before, in *jobs* where many valid patterns are found by *FP-Growth*, these two steps significantly impact the overall performance, as they are currently executed sequentially on a single CPU core in contrast to the parallel *FP-Growth*. This is also the primary reason for the weaker scaling when the number of neurons increases, which can lead to a significant increase in found patterns. Concluding, it can be said that although our implementation scales not as well as the original, it still scales adequately even when confronted with long data sets or ones containing several hundred neurons. Furthermore, due to the overall significantly lower runtime, our proposed flow is between one and two orders of magnitude faster than the original.

## 4. Discussion

Finding spike patterns in parallel spike trains using the *FP-Growth* pattern mining algorithm and a custom filter function is one of the most time-intensive parts of the *SPADE* method. In the currently available implementation, pattern mining is performed using a C-based Python module, while the filtering is done directly in Python. There are some significant flaws in the current flow that result in a significantly increased runtime. On the one hand, all found patterns need to be converted from C to Python, which takes a long time and consumes a large amount of memory. On the other hand, performing the pattern filtering in the Python programming language also negatively affects the runtime. Therefore, in this work, we developed a multi-threaded C++-based Python module that, while maintaining the original flow's functionality, performed the task between 27 and 200 times faster, while at the same time being 67 to 280 times as energy efficient depending on the executing hardware. By integrating the pattern filtering function directly into the *FP-Growth* implementation developed in this work, we dramatically reduced the number of produced patterns that need to be converted to Python. This reduced not only the runtime but also the memory consumption. Furthermore, we integrated multi-threading and distributed computing capabilities into our *FP-Growth* implementation to fully utilize the CPU of one or more compute nodes. Additionally, we showed that our implementation scales reasonably when the number of neurons or the length of the recording is changed and is able to finish the processing of a very large data set (1,003 s of neuron activity) in less than an hour, a task that was not possible using the original version. As a result, the improvement of the method enables the analysis of experimental data in a feasible amount of time together with the statistical evaluation of mined patterns, i.e., in the case where *FP-Growth* is applied not only on the original data set, but also on its surrogates, as explained in section 2.3. Our optimized flow opens up the possibility to perform more complex analyses due to the highly reduced amount of time. This makes it possible to handle large state-of-the-art data sets, such as data recorded from multiple Utah arrays (Chen et al., 2020), or Neuropixel probes (Juavinett et al., 2019), and to combine the results of SPADE with other approaches to investigate the correlative structure of neuronal dynamics (Diana et al., 2019; Watanabe et al., 2019; Williams et al., 2020).

### 4.1. Platform Comparison

**Figure 6:**
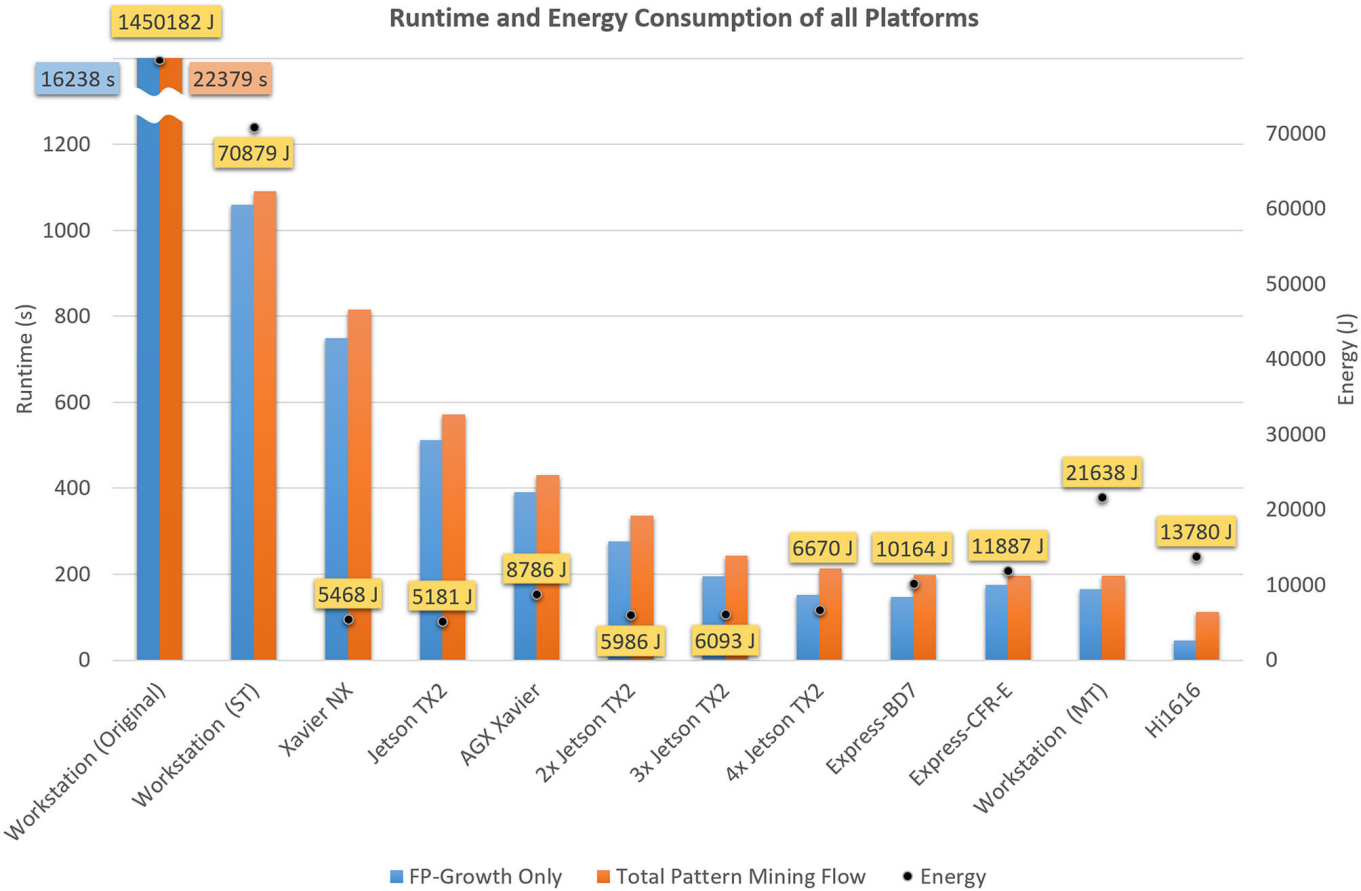
Runtimes of the optimized flow on all considered platforms (cf. Table 7). *MT* refers to the multi-threaded, *ST* to the single-threaded and *Original* to the baseline (currently used) version. A detailed overview over the different platforms and their features is presented in Table 9.

Here, we perform a concluding comparison of the results achieved by our optimized implementation on the different platforms. The performance, in terms of runtime, memory consumption, and energy efficiency of our implementation was evaluated on a workstation system, a *Hi1616* microserver equipped with two HiSilicon *Hi1616* CPUs, an *ADLINK Express-BD7* equipped with an Intel Xeon D-1577, an *ADLINK Express-CFR-E* equipped with an Intel Xeon E-2276 and three different embedded computing devices from NVIDIA, namely *Xavier NX, AGX Xavier* and *Jetson TX2*. For an easy comparison, some of the most distinctive features of each platform focused on the respective CPU are shown in Table 9. These are, among others, the architecture, TDP^22^, and ISA^23^ of the CPU, as well as the type of memory installed. Figure 6 shows the performance, in terms of *FP-Growth* runtime only, total execution time, and energy consumption, of the different platforms. The graph is sorted by total execution time. *Total Pattern Mining Flow* refers to the time required for the entire accelerated flow, i.e., *FP-Growth*-based pattern mining, pattern filtering, closed detection, and data conversion to Python, while *FP-Growth Only* exclusively shows the time required for the *FP-Growth*-based pattern mining and the pattern filtering.

**Table 9:** Overview of the different platforms and their distinctive features.

**Platform**	**CPU**	**Architecture**	**Memory**	**Cores**	**Threads**	**Clockrate**	**TDP**	**ISA**
Workstation	E5-1650 v4	Haswell	DDR4	6	12	3.6 GHz	140 W	x86
Exp.-BD7	D-1577	Broadwell	DDR4	16	32	1.3 GHz	45 W	x86
Exp.-CFR-E	E-2276ME	Coffee Lake	DDR4	6	12	2.8 GHz	45 W	x86
Hi1616	Hi1616	Kunpeng	DDR4	32	32	2.4 GHz	85 W	A64
Jetson TX2	Cortex-A57	ARMv8-A	LPDDR4	4	4	2.0 GHz	7.5 W	A64
	Denver	Denver	LPDDR4	2	2	2.0 GHz	7.5 W	A64
AGX Xavier	Carmel	Carmel	LPDDR4X	8	8	2.0 GHz	30 W	A64
Xavier NX	Carmel	Carmel	LPDDR4X	6	6	1.4 GHz	15 W	A64

Except for the *Hi1616* microserver, *FP-Growth* consumed the largest portion of the runtime on all platforms. Thanks to its 64 cores, the *Hi1616* microserver achieved the highest parallel processing performance and overall fastest execution time (111 s). However, due to the low individual core performance, a significant amount of time was required for the flow's sequential parts, which noticeably increased the full runtime. This, in turn, affected the power consumption, which resulted in the third-highest energy consumption (13,780 J). As can be expected, the longest runtime (1,091 s) and the highest energy consumption (70,876 J) belong to the single-threaded version's execution on the workstation. However, these values are still one order of magnitude lower than the original implementation, whose results are 20 times higher in both aspects (22,379 s and 1,450,185 J).

For identifying the most suitable platform for the given application, both runtime, and energy consumption have to be considered. The lowest energy consumption was obtained using one *Jetson TX2* (5,181 J), while the fastest runtime was achieved on the *Hi1616* microserver (111 s). Comparing the two in consideration of the respective other value, the *TX2* takes five times longer, while the *Hi1616* microserver consumes about 2.7 times more energy. When focusing on only one of these values, it is straightforward to choose the most suitable platform. However, when both factors are of equal importance, the decision becomes significantly more challenging. The most balanced ratio between runtime and energy consumption was achieved on the platforms we looked at when two or three *Jetson TX2* were used in parallel.

### 4.2. Summary and Future Work

We have presented our optimized version of the *SPADE* method's pattern mining flow in this work, using a custom-tailored *FP-Growth* implementation. Using a data set containing spike trains from experimental data, we performed our evaluation on a typical *SPADE* use case. We showed how our implementation handles different input settings by varying the parameter configuration for the minimum size and occurrence number. Furthermore, using our distributed approach on up to four TX2, a near-linear scaling for the part computed in parallel, i.e., the *FP-Growth*-based pattern mining, was achieved. In this work, our primary focus was the acceleration of the pattern mining and result filtering tasks, as they account for between 85 and 90% of the overall runtime. On the one hand, we showed that our improved version was, depending on the platform used, between 27 and 200 times faster compared to the original implementation. On the other hand, all platforms' energy consumption was up to two orders of magnitude lower than the original *FP-Growth* version currently used in *SPADE*. The highest energy efficiency was achieved by the embedded devices, which, when executing our flow, required only between 41% and 24% of the energy consumed by the workstation, running the multi-threaded version of our optimized implementation. Furthermore, the execution on embedded devices is now possible; previously, this was prevented by the high memory requirements.

In the future, we intend to further improve our flow by looking at ways to accelerate the sections currently executed sequentially, i.e., the closed pattern detection and the data conversion to Python. Depending on the number of patterns found by *FP-Growth*, these parts become the bottleneck, as mentioned in section 3.5. An example of this can be seen in *job 2*, where, depending on the platform, these tasks account for a significant portion of the job's and the overall runtime. For this reason, we will be looking into implementations for parallel closed pattern detection, e.g., the propositions made by Lucchese et al. (2007) and Huynh et al. (2017). Besides the acceleration of these sections, we plan to integrate the filtering even deeper into our *FP-Growth* implementation, e.g., by marking all items that reside in the first bin of their respective windows. This could enable even faster filtering and, in addition, might also reduce the number of header table entries to check. Furthermore, we want to evaluate the usability of GPU-based *FP-Growth* and closed pattern detection implementations, like the ones described in Wang and Yuan (2014), Jiang and Meng (2017), and Wu et al. (2019). At the same time, it will also be of interest to analyze the applicability of a heterogeneous CPU and GPU implementation, i.e., where the workload is shared between the CPU and the GPU. This is something from which especially the embedded devices could significantly benefit, as their GPU is directly connected to the DDR memory allowing for fast data exchange. We also intend to further improve our distributed computing setup performance by exploring different strategies like the ones proposed by Li et al. (2008) and Chen et al. (2009). Additionally, we suggest to investigate different pattern mining algorithms, e.g., LCM^24^ (Uno et al., 2004) or DPT^25^ (Qu et al., 2020), and evaluate their performance in the given use case. Finally, we want to analyze further the *SPADE* code surrounding *FP-Growth* to find more potential improvement points. At the same time, it might be worthwhile to analyze the *SPADE* code as a whole and identify bottlenecks that can be accelerated using custom C/C++ modules.

## Data Availability Statement

Publicly available datasets were analyzed in this study. This data, together with the source code for the Python module presented in this paper can be found at: https://github.com/fporrmann/FPG. The accelerated version of the SPADE method presented in this article is included in the Elephant^26^ GitHub project at: https://github.com/NeuralEnsemble/elephant and will be featured starting from the official release 0.11.0. For an interactive demonstration on how to use the SPADE method, please refer to the tutorial page of the Elephant documentation available at: http://tutorials.python-elephant.org.

## Author Contributions

FP: conceptualization, software implementation and optimization, testing, evaluation, and writing original draft preparation. SP and FP: visualization. AS: data preparation. FP, SP, AS, AK, MD, JH, and UR: writing review and editing. JH and UR: project administration and funding acquisition. All authors have read and agreed to the published version of the manuscript.

## Funding

This publication was supported by the VEDLIoT and LEGaTO projects, which received funding from the European Union's Horizon 2020 research and innovation program under grant agreements Nos. 957197 and 780681. Besides, the work was supported by the PhD program Design of Flexible Work Environments–Human-Centric Use of Cyber-Physical Systems in Industry 4.0, funded by the North Rhine-Westphalian funding scheme Forschungskolleg and affiliated to the Research Institute for Cognition and Robotics (CoR-Lab), Bielefeld University. This project has also received funding from the European Union's Horizon 2020 Framework Programme for Research and Innovation under Specific Grant Agreement No. 945539 (Human Brain Project SGA3) and the Helmholtz Association Initiative and Networking Fund under project number ZT-I-0003 and the VSR computation grant JINB33, Jülich.

## Conflict of Interest

The authors declare that the research was conducted in the absence of any commercial or financial relationships that could be construed as a potential conflict of interest.

## Publisher's Note

All claims expressed in this article are solely those of the authors and do not necessarily represent those of their affiliated organizations, or those of the publisher, the editors and the reviewers. Any product that may be evaluated in this article, or claim that may be made by its manufacturer, is not guaranteed or endorsed by the publisher.

## Footnotes

^1^**S**pike **PA**ttern **D**etection and **E**valuation.

^2^ http://python-elephant.org

^3^**F**requent **P**attern Growth.

^4^**F**requent **P**attern Tree.

^5^ http://spark.apache.org/

^6^ https://borgelt.net/pyfim.html

^7^Open Multi-Processing - https://www.openmp.org/.

^8^**M**essage **P**assing **I**nterface.

^9^**R**esource-**E**fficient **C**luster **S**erver – https://embedded.christmann.info/products.

^10^**F**ield **P**rogrammable **G**ate **A**rray.

^11^**S**ystem-**o**n-a-**C**hip.

^12^ https://www.picmg.org/openstandards/com-express/

^13^ https://www.adlinktech.com/Products/Computer_on_Modules/COMExpressType7/Express-BD7

^14^ https://www.adlinktech.com/Products/Computer_on_Modules/COMExpressType6/Express-CFR

^15^ https://www.tek.com/oscilloscope/mdo4054b-6

^16^ https://www.tek.com/datasheet/30-ac-dc-current-probe

^17^Watt.

^18^Joule (watt-second).

^19^ https://developer.nvidia.com/embedded/jetson-agx-xavier-developer-kit

^20^ https://developer.nvidia.com/embedded/jetson-xavier-nx

^21^ https://developer.nvidia.com/embedded/jetson-tx2

^22^**T**hermal **D**esign **P**ower.

^23^**I**nstruction **S**et **A**rchitecture.

^24^**L**inear time **C**losed itemset **M**iner.

^25^**D**ynamic **P**refix **T**ree.

^26^RRID:SCR003833; python-elephant.org.
